# Identification of prognostic and cellular senescence gene E2F1 of papillary thyroid carcinoma through bioinformatics analyses and experimental verification

**DOI:** 10.3389/fgene.2025.1605385

**Published:** 2025-09-18

**Authors:** Bin Yu, Shu-Yan Zhao, Yun-Hua Zhu, Jun-Jie Luo, Ke Zheng, Bin-Jie Shen, Yi-Lin Shen, Huan-Xin Zhong

**Affiliations:** ^1^ Thyroid & Breast Surgery, The Second Affiliated Hospital of Zhejiang University School of Medicine, Linping Campus, Hangzhou, Zhejiang, China; ^2^ Department of Thyroid Surgery, The First Affiliated Hospital of Kunming Medical University, Kunming, Yunnan, China; ^3^ Gynecology Department, The Second Affiliated Hospital of Zhejiang University School of Medicine, Linping Campus, Hangzhou, Zhejiang, China

**Keywords:** cell senescence, thyroid cancer, E2F1, prognosis, immunotherapy

## Abstract

**Objective:**

This work aimed to find a new prognostic cell senescence gene to predict the prognosis of patients with papillary thyroid carcinoma (PTC).

**Methods:**

The data of the patients with PTC were collected from the Cancer Genome Atlas (TCGA) database. The gene set of cellular senescence was collected from the website of CellAge. The function of hub genes was analyzed by various bioinformatics methods including expression analysis, survival analysis, and nomogram analyses. Real-time quantitative PCR, cell transfection, colony formation assay, Western blot, wound healing assay, transwell assay, cell counting Kit-8, flow cytometry, and immunohistochemistry staining were performed to verify the function of hub gene.

**Results:**

E2F1 was finally screened as the key senescence gene, and its expression was higher in PTC tumors than in normal. KM curve indicated that PTC patients with higher expression of the E2F1 had longer survival times. The GSEA showed that the high expression group of E2F1 was enriched in DNA replication and so on. Cell experiments showed that overexpression of E2F1 significantly increased relative protein expression of senescence related markers, including p21, p53, γ-H2AX, and p16INK4a. Cell experiments also showed that overexpression of E2F1 inhibited the invasion, proliferation, and migration of tumor cells. While knockdown of E2F1 reversed these results.

**Conclusion:**

E2F1 was found to be upregulated in PTC, with its high expression significantly correlated to a favorable patient prognosis. E2F1 suppresses malignant tumor phenotypes by modulating cellular senescence. A predictive model integrating E2F1 and clinical features accurately forecasts poor prognosis, indicating E2F1’s potential as a therapeutic target for PTC.

## 1 Introduction

Thyroid cancer (THCA) is a common cancer with high prevalence in human endocrine diseases around the world. It was already the seventh most common cancer, with more than 821 thousand, new cases of THCA based on the Global Cancer Statistics 2022 (Bray et al., 2024). The occurrence of THCA has been increasing annually. According to the reported literature, there were 29.4 individuals per 100 thousand in the United States (Gudmundsson et al., 2017). Papillary thyroid carcinoma (PTC) accounts for approximately 80%–90% of all THCA. Histologically, the tumor cells exhibit papillary architecture with fibrovascular cores, characteristic ground-glass nuclei containing nuclear grooves and pseudoinclusions, and frequent stromal calcifications. Although typically indolent with a favorable prognosis, PTC’s substantial disease burden warrants attention, particularly since 10%–15% of cases represent the moderately differentiated PTC variant demonstrating aggressive clinical behavior and high mortality rates (Ambrosi et al., 2017). Consequently, elucidating PTC’s molecular mechanisms and identifying reliable prognostic biomarkers hold significant clinical importance for optimizing patient management.

Cell senescence arrests the cell cycle stably, which inhibits the proliferative life span stably (Calcinotto et al., 2019). Cells have different features related to gene expression, substance metabolism, cytokine secretion and other factors (Fridman and Tainsky, 2008). As one of the key processes in tumorigenesis, cell senescence can not only play a tumor suppressor role through persistent cell cycle arrest, but also promote cancer by senescence-associated secretory phenotype (SASP) -mediated inflammation, matrix remodeling and immune reprogramming, showing a ‘double-edged sword’ effect (Feng et al., 2025; Wang et al., 2020). In THCA, most studies have shown that the development of THCA is related to cell senescence. Early studies have shown that the introduction of oncogenes associated with THCA (such as RAS) into human primary thyroid cells can induce typical oncogene-induced senescence (OIS) phenotypes, including SA-β-gal positive, p16 (INK4A)/p21 (CIP1) upregulation and SAHF formation, suggesting that OIS is one of the natural barriers to malignant transformation of thyroid epithelium (Vizioli et al., 2011; Vizioli et al., 2014). Studies have shown that senescent tumor cells are enriched in the ' invasion front ' of PTC, and drive the collective invasion of CXCR4+ cancer cells by forming CXCL12 gradient, while improving the viability of tumor cells in lymphatic vessels and lymph nodes, revealing the active role of senescent cells in the metastasis cascade (Kim et al., 2017). In the tumor microenvironment, senescent thyroid cells and tumor cells can induce the polarization of human monocytes to M2-like macrophages through PGE2-mediated paracrine effects and enhance immunosuppression. The co-localization of cancer-associated fibroblasts (CAFs) and senescent thyroid cells at the forefront of tumor invasion also histologically supports the ‘aging-matrix-immune’ axis involved in PTC progression (Mazzoni et al., 2019; Minna et al., 2020). Some studies have proposed a variety of senescence-related genes models based on the aging-related molecular map in TCGA/GEO. For example, the three-gene model constructed with E2F1, SNAI1, and PLA2R1 showed good survival discrimination and immune infiltration in PTC (Wen and Guo, 2023). Six-gene features (ADAMTSL4, DOCK6, FAM111B, SEMA6B, MRPS10, PSMB7) can stably distinguish high-and low-risk subgroups, and indicate immune checkpoint expression, immunotherapy response, and drug sensitivity differences in THCA (Hong et al., 2023).

However, there is still a lack of systematic research on the dual integration of cellular senescence and clinical outcome. This study aims to systematically identify and verify the key genes and characteristics associated with PTC prognosis and cell senescence, utilizing large cohort bioinformatics analysis and *in vitro* experimental verification. The goal is to provide actionable biomarkers and potential targets for risk stratification and precision treatment.

## 2 Materials and methods

### 2.1 Data source

Transcription data and corresponding clinical data of the patients with PTC were collected from the Cancer Genome Atlas (TCGA, https://www.cancer.gov/about-nci/organization/ccg/research/structural-genomics/tcga) database. The data source included 502 patients with PTC and 48 normal individuals. The transcription data were normalized by the “limma” package of the R software. The gene set of cellular senescence was collected from the website of CellAge (https://genomics.senescence.info/cells/), a database of genes associated with cell senescence. Data collated by the developers of this website is based on gene manipulation experiments in different human cell types. A gene expression signature of cellular senescence is also available from the database (Avelar et al., 2020). The cellular senescence gene set included 279 genes (Supplementary Table S1).

### 2.2 Screening of prognostic genes associated with cellular senescence

First, we compared the expression levels of 279 cellular senescence genes between normal individuals and patients with PTC. Genes with p < 0.05 and |log2 (fold change) | > 1 were considered differentially expressed cellular senescence genes (DECSG). Then, univariate analysis was used to explore the relationship between the 279 senescence genes and the prognosis of PTC patients. Genes with p < 0.05 were considered the prognostic cellular senescence genes (PCSG). Subsequently, the overlapped genes between the DECSG gene set and PCSG gene set were obtained by package “Venn” of the “R” software. Last, the multivariable Cox regression analysis was performed based on the overlapped genes. In multivariate Cox regression analysis, a gene with p < 0.05 was considered as a key prognostic cellular senescence gene.

### 2.3 The expression of key prognostic cellular senescence gene

The key prognostic cellular senescence gene expression was compared between normal individuals and patients with PTC using T-test. The paired t-test was used to analyze the expression between the PTC tissue and paracancerous tissue of patients with PTC. Moreover, the protein expression detected by immunohistochemistry in tumor and normal tissue was explored from the Human Protein Atlas (HPA) (https://www.proteinatlas.org/). HPA is a comprehensive resource for exploring the human proteome, which includes antibody-based tissue microarray analysis and a large amount of proteomic and transcriptomic data. In addition, we verified the expression of key prognostic cellular senescence genes through the GSE3467 dataset and GSE33630 dataset from the Gene Expression Omnibus (GEO) (https://www.ncbi.nlm.nih.gov/geo/) database.

### 2.4 Survival analysis

Kaplan–Meier (KM) curves were plotted based on overall survival (OS) and the gene expression. The optimal cut-off point of KM survival curves was generated through the “res. cut” function in the “survminer” package and the log-rank test was used to analyze the differences between survival curves. The receiver operating characteristic (ROC) curve was constructed to assess the prediction in differentiating patients with PTC from normal individuals. Additionally, the univariate and multivariable Cox regression analysis was used to explore the association between clinical features and the patient’s OS for determining the effect of the expression of hub genes in patients with PTC. Last, the R package “rms” was utilized to build the nomogram. Decision analysis curve (DCA), ROC, and calibration curves were conducted to evaluate the predictive performance of the nomogram model.

### 2.5 Gene set enrichment analysis

Gene Set Enrichment Analysis (GSEA) was initially performed to rank the ordered list of differentially expressed genes (DEGs) in this study. GSEA was conducted to assess the significant survival differences observed between the high-expression and low-expression patient groups in PTC. The c2. cp.kegg.v7.4. symbols.gmt dataset was downloaded from the Molecular Signatures Database and used to evaluate relevant pathways and molecular mechanisms. Set permutations were performed 1,000 times for each analysis. P value of <0.05 and an FDR of <0.25 were considered statistically significant.

### 2.6 Immune microenvironment correlation analysis

To research the relationship between the expression of the hub gene and six kinds of immune cells, we conducted the analysis of immune cell infiltration levels by the Tumor Immune Estimation Resource (TIMER) methods based on the TIMER2.0 database (http://timer.cistrome.org/) which was widely used to study immune cell infiltrates across various tumors (Li et al., 2020). The related immune cells included B cells, CD8+T cells, CD4+T cells, macrophages, neutrophils, and dendritic cells, and the relationship was corrected according to tumor purity. Moreover, the patient with PTC was grouped into two groups by the expression level of the hub gene to research further correlations between the hub gene and the immune microenvironment score of six kinds of immune cells.

### 2.7 Cell culture

Normal thyroid cell lines (Nthy-ori-3-1) and thyroid cancer cell lines (TPC-1 and K1) were cultured in DMEM supplemented with 20% fetal bovine serum (FBS) and maintained at 37 °C with 5% CO_2_.

### 2.8 Real-time quantitative PCR

The mRNA levels of the target genes were assessed following the manufacturer’s protocols. In summary, total RNA was extracted from cell samples using TRIzol reagent (Sigma, United States), and its concentration was measured using the Nanodrop 100 spectrophotometer (Thermo, United States). Complementary DNA (cDNA) was synthesized using the Hiscript QRT SuperMix (Vazyme, China). The qPCR reaction (10 µL) was conducted with the SYBR Green Master Mix Kit (Vazyme, China) and analyzed using the Biosystems 7,500 Sequence Detection system. The RT-qPCR cycling conditions were set as follows: initial denaturation at 95 °C for 30 s, followed by 40 cycles of 95 °C for 5 s, annealing at 60 °C for 30 s, and finishing with 1 cycle of 95 °C for 15 s, 60 °C for 30 s, and 95 °C for 15 s. Each assay was carried out in triplicate. The gene expression was normalized to GAPDH, and the relative expression of the genes was calculated using the 2^−ΔΔCT^ method. The primer sequences of E2F1:forward 5′-TGA​ATC​TGA​CCA​CCA​AGC​G-3′; reverse 5′-TTC​TGC​ACC​TTC​AGC​ACC​TC-3′, GAPDH: forward 5′-GGA​GCG​AGA​TCC​CTC​CAA​AAT-3′; reverse 5′-GGC​TGT​TGT​CAT​ACT​TCT​CAT​GG-3′.

### 2.9 Cell transfection

We used small interfering RNA (siRNA) to silence the E2F1 gene in K1 cells. The specific steps were as follows: 1) siRNA was briefly centrifuged and prepared into a 20 µM stock solution using RNase-free H_2_O. 2) 24 h before transfection, 0.5 - 2 × 10^5^ cells were seeded, and the cell confluence at the time of transfection was 50%. 3) For each well in a 24-well plate, 25 µL of antibiotic-free and serum-free DMEM medium was used to dilute 1.25 µL of siRNA and 500 ng of plasmid DNA. The mixture was gently pipetted 3 - 5 times to ensure even mixing, followed by the addition of 1.0 µL of EpFed transfection reagent. The solution was again gently pipetted 3 - 5 times to ensure thorough mixing. The final siRNA concentration in the cells was 50 nM, and the mixture was incubated at room temperature for 20 min 4) The transfection mixture (siRNA/plasmid-DNA and transfection reagent) was evenly added to each well of the 24-well plate. The plate was gently rocked back and forth to ensure even distribution of the mixture. 5) The cell plate was then incubated at 37 °C with 5% CO_2_ for 72 h. Additionally, an amplification sequence targeting E2F1 was synthesized and inserted into the pcDNA3.1 vector to generate an overexpression of E2F1 in TPC-1 cell. TPC-1 cells were cultured for 24 h and then transfected with vector, or vector-E2F1. Transfection was carried out using Lipofectamine^®^ 3,000 reagent (Invitrogen) and incubated at 37 °C for 48 h. RT-PCR and Western blot assays were used to detect the transfection effect.

### 2.10 Colony formation assay

The colony formation assay was used to assess the survival ability of cells *in vitro*. The steps are as follows: Cell preparation: Cells or pre-treated cells were digested with trypsin and collected, then resuspended in a complete medium to obtain a single-cell suspension. The cell concentration was counted. Cell seeding: The cell suspension was adjusted to a concentration of 1,000 cells/mL. Then, 1,000 cells were seeded per well in a 6-well plate. Cell culture: The cells were cultured in a cell incubator for 1–3 weeks, with medium changes every 3 days and periodic observation of cell status. Fixation and staining: After culture, cells were washed with PBS, then fixed with 1 mL of 4% paraformaldehyde for 30–60 min. After washing again, 1 mL of crystal violet staining solution was added to each well and incubated for 10–20 min. Colony counting: Colonies were counted under a microscope, or images of the entire 6-well plate and each individual well was taken for documentation. Data analysis: Colony formation counts from each group were processed using ImageJ software to evaluate the number of colonies formed in each group. This can be used to assess cell survival or proliferation changes compared to the control group.

### 2.11 Protein extraction and Western blot

Proteins were extracted from tumor cells using RIPA lysis and extraction buffer (KeyGen Biotechnology, Nanjing, China). The protein concentration was determined using the BCA method. Equal amounts of protein were separated by SDS-PAGE and transferred onto a PVDF membrane (EMD Millipore, Burlington, MA). The membrane was then blocked with 5% (v/v) Bovine Serum Albumin (BSA) for 1 h. The membrane was incubated overnight at 4 °C with an appropriately diluted primary antibody (E2F1 antibody, 1:800; Biorbyt, England). Afterward, the membrane was incubated with the secondary antibody at room temperature for 1 h. Protein bands were detected using the ECL chemiluminescence kit (Thermo Fisher Scientific). Finally, the protein bands were analyzed semi-quantitatively using ImageJ (National Institutes of Health, Bethesda, MD, United States). The results are presented as the ratio of the optical density (OD) of the target protein to the internal control.

### 2.12 Wound healing assay

The scratch assay is used to study cell migration and repair behavior *in vitro*. The specific procedure is as follows: 1) Before seeding cells, mark horizontal lines on the bottom of a 12-well plate using a marker pen. 2) Digest the cells, then terminate the digestion and centrifuge at 1,500 rpm for 3 min. Discard the supernatant, wash the cell pellet once with PBS, and perform cell counting. 3) After counting the cells, resuspend them and seed them into the 12-well plate, aiming for a confluency that ensures the plate bottom is covered once the cells adhere. 4) Once the cells have adhered, use a 200 μL pipette tip to create scratches in the cell monolayer, keeping the pipette tip perpendicular to the plate to ensure uniform scratch widths. 5) Aspirate the culture medium and wash the plate three times with PBS to remove cell debris generated by the scratching process. 6) Add serum-free culture medium and capture images to document the initial condition. 7) Place the plate in an incubator and capture images every 4–6 h 8) Analyze the experimental results by comparing the collected images over time.

### 2.13 Transwell assay

Cells were digested with trypsin and resuspended in cell suspension (80,000 cells per well), then seeded into the upper chamber of a Transwell insert (24-well, 8 µm pore size) (Corning) with 100 µL of cell suspension. In the lower chamber, 500 µL of DMEM supplemented with 30% FBS was added. After 24 h of incubation at 37 °C, migrating cells on the underside of the polycarbonate membrane were fixed with 4% pre-cooled paraformaldehyde for 30 min. The cells were then stained with 0.1% crystal violet for 20 min at room temperature. After washing with PBS, five random fields were selected under a fluorescence microscope (200 × magnification, Olympus), and the migration rate was calculated based on the number of migrating cells.

### 2.14 Cell counting Kit-8

First, cells were cultured in standard cell culture flasks and monitored for growth under a microscope. When the cell density reached 80%–90%, cells were digested with 0.25% Trypsin-EDTA. Fresh DMEM medium was added, and the cell suspension was thoroughly mixed. Then, 100 µL of the cell suspension was seeded into each well of a 96-well plate. The plate was cultured in a 37 °C incubator for 12, 24, 36, and 72 h. Next, the cell proliferation and toxicity assay were performed. Specifically, 10 µL of CCK-8 reagent was added to each well, and the plate was incubated for an additional 4 h in the incubator. Finally, the 96-well plate was removed, and the absorbance at 490 nm (OD490) was measured using a microplate reader. The cell proliferation curve was then plotted based on the OD490 values.

### 2.15 Immunohistochemistry (IHC) staining

Formalin-fixed PTC and paired normal tissues were deparaffinized in xylene and rehydrated in ethanol solutions. To block endogenous peroxidase activity and nonspecific binding sites, the tissues were incubated with 3% hydrogen peroxide. Next, the tissues were incubated overnight at 4 °C with the primary antibody (anti-E2F1, diluted 1:2,500), followed by a 1-hour incubation at room temperature with the secondary antibody, HRP-conjugated goat anti-rabbit IgG. The tissue was then stained with 3,3′-diaminobenzidine (DAB) solution for 3 min. The cell nuclei were counterstained with hematoxylin. Tissue sections were examined with a Nikon Eclipse model N i-U microscope.

### 2.16 Flow cytometry

Single cell suspensions of tumors were analyzed by flow cytometry. Treated cell suspensions were collected and centrifuged (300 × g, 5 min, 4 °C). After discarding the supernatant, cells were washed once with ice-cold phosphate-buffered saline (PBS). Subsequently, cells were resuspended in β-galactosidase staining buffer (pH 6.0) containing 20 μM C12FDG and incubated at 37 °C in a CO_2_-free environment for 90 min, protected from light. During incubation, the highly active β-galactosidase in senescent cell lysosomes hydrolyzes C12FDG, liberating fluorescein to generate fluorescent signals. Immediately after incubation, cells were washed twice with ice-cold PBS to quench the reaction and remove unreacted substrate. Cells were fixed briefly with 4% paraformaldehyde (4 °C, 10 min). Finally, cells were resuspended in ice-cold PBS containing 1% paraformaldehyde. Flow cytometry analysis was performed using a flow cytometer, and data acquisition was conducted with FlowJo software (Version 10; FlowJo, LLC).

### 2.17 Statistical analysis

The qualitative data between groups were compared with a rank-sum test using IBM SPSS 21.0 software (IBM SPSS, Armonk, NY, United States) as the data did not conform to a normal distribution. In addition, data visualization was performed using SangerBox 3.0 (http://vip.sangerbox.com/home.html) (Shen et al., 2022). The bilateral p values < 0.05 were considered statistically significant.

## 3 Results

### 3.1 Identification of prognostic genes associated with cellular senescence

**FIGURE 1:**
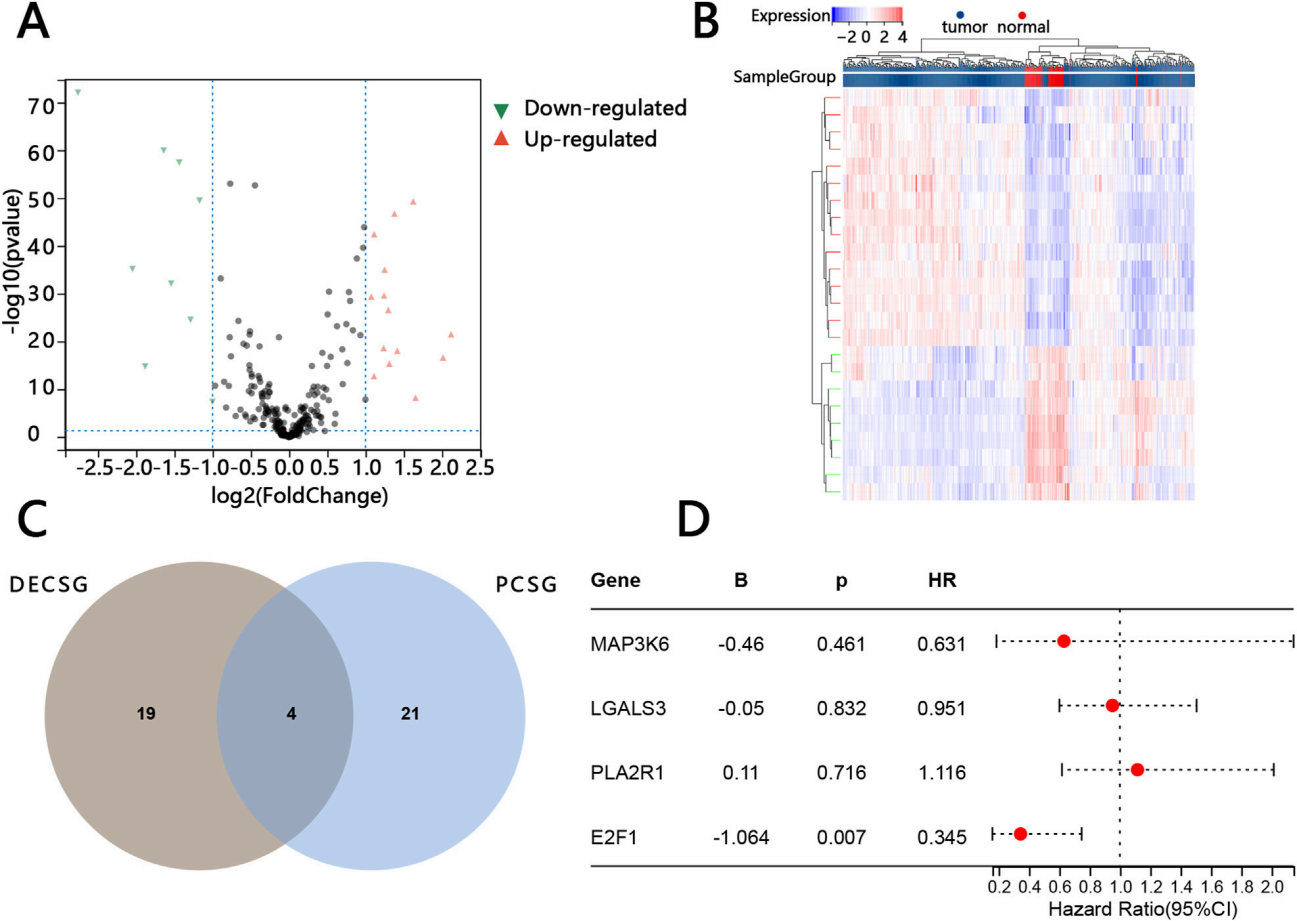
Screening of key prognostic-related cellular senescence genes. Volcano map of differentially expressed cellular senescence genes (DECSG) between the normal tissue and PTC tissue **(A)**. Heatmap of DECSG **(B)**. The overlapped genes between the DECSG and prognostic cellular senescence genes PCSG **(C)**. The multivariate cox regression analysis of the overlapped genes **(D)**.

Twenty-three DECSG between normal individuals and patients with PTC were found via the “limma” R package (p value <0.05 and |log2FC| > 1) (Supplementary Table S2). DECSG, related to tumorigenesis, included 15 upregulated genes and 8 downregulated genes (Figure 1A). Those DECSGs were plotted into the heatmaps (Figure 1B). Twenty-five PCSG, related to prognosis, were screened by univariate Cox regression analysis based on P < 0.05 (Supplementary Table S3). Subsequently, the 4 overlapped genes (MAP3K6, LGALS3, PLA2R1, E2F1) were obtained by package “Venn” of the “R” software (Figure 1C). E2F1 was finally screened by the multivariable Cox regression analysis based on the overlapped genes as only E2F1 was independently related to the prognosis of patients (Figure 1D).

### 3.2 Clinical baseline

To avoid variability between sample groups, patients with PTC were divided into two groups based on the expression of E2F1. The baseline data of patients is shown in Table 1, which indicates that no variable showed statistical significance between the two groups.

**TABLE 1:** The correlation between E2F1 expression and clinical characteristics of patients.

Characteristics	High expression	Low expression	p
Sex, n (%)			0.47
Female	243 (48.41%)	124 (24.70%)	
Male	84 (16.73%)	51 (10.16%)	
T, n (%)			0.11
T1	82 (16.33%)	61 (12.15%)	
T2	108 (21.51%)	56 (11.16%)	
T3	122 (24.30%)	48 (9.56%)	
T4	14 (2.79%)	9 (1.79%)	
TX	1 (0.20%)	1 (0.20%)	
N, n (%)			0.45
N0	148 (29.48%)	81 (16.14%)	
N1	150 (29.88%)	73 (14.54%)	
NX	29 (5.78%)	21 (4.18%)	
M, n (%)			0.32
M0	183 (36.53%)	99 (19.76%)	
M1	8 (1.60%)	1 (0.20%)	
MX	136 (27.15%)	74 (14.77%)	
Stage, n (%)			0.57
I	183 (36.60%)	98 (19.60%)	
II	30 (6.00%)	22 (4.40%)	
III	76 (15.20%)	36 (7.20%)	
IV	38 (7.60%)	17 (3.40%)	
Age, (years)	47.08 ± 15.49 (65.14%)	47.84 ± 16.48 (34.86%)	0.24
Neoplasm depth, (cm)	1.76 ± 0.93 (67.25%)	1.69 ± 0.88 (32.75%)	0.49
Neoplasm length, (cm)	2.94 ± 1.71 (67.25%)	2.84 ± 1.61 (32.75%)	0.86
Neoplasm width, (cm)	2.30 ± 1.29 (67.25%)	2.19 ± 1.16 (32.75%)	0.61

### 3.3 Expression of E2F1 in PTC

**FIGURE 2:**
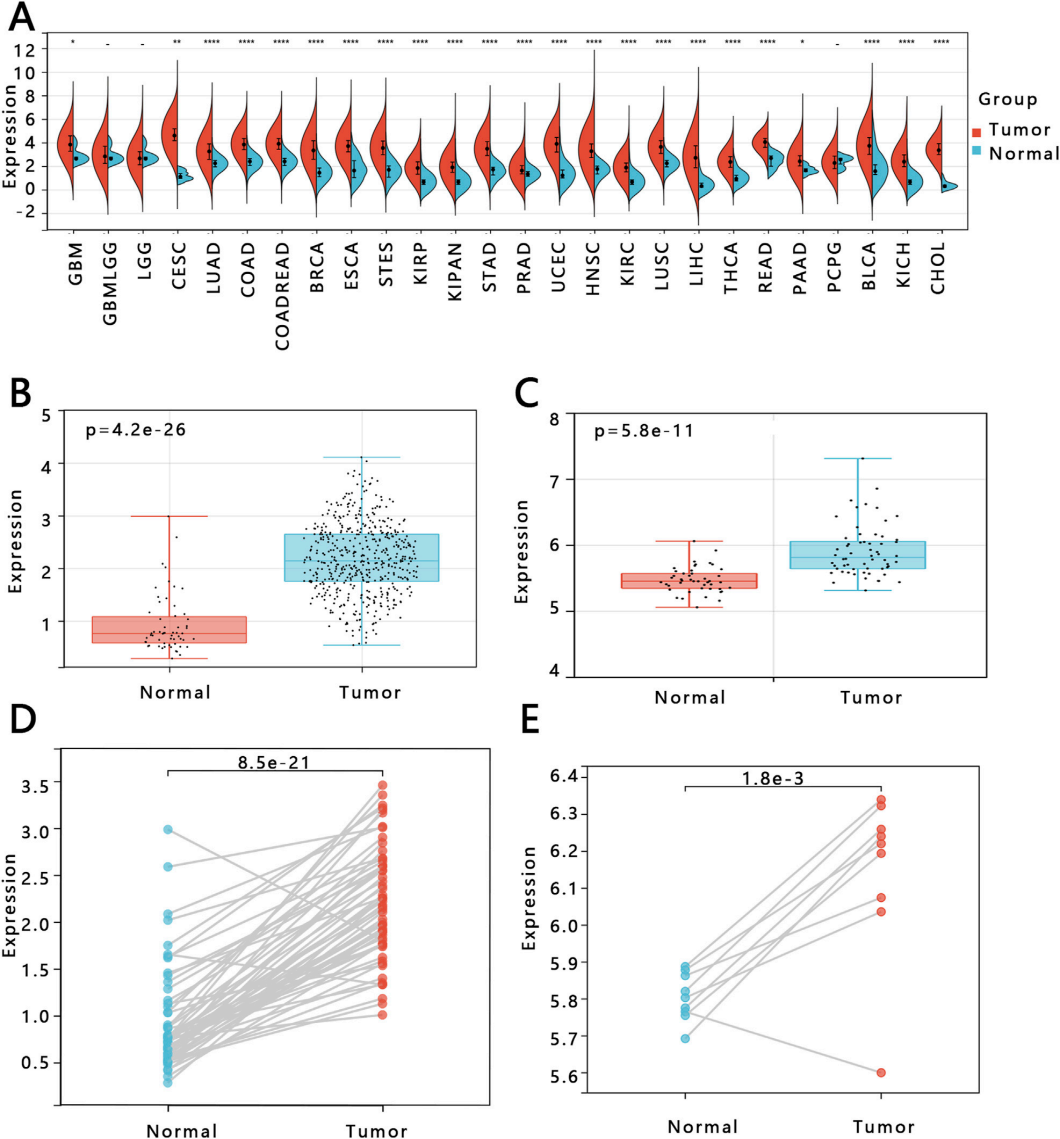
Exploration of E2F1 gene expression. The expression analysis of E2F1 mRNA in pan-cancer **(A)**. Comparison between PTC tissue and normal thyroid tissue both in TCGA-PTC database **(B)** and GSE33630 dataset **(C)**. Comparison between paired samples both in TCGA-THCA database **(D)** and GSE3467 dataset **(E)**.

**FIGURE 3:**
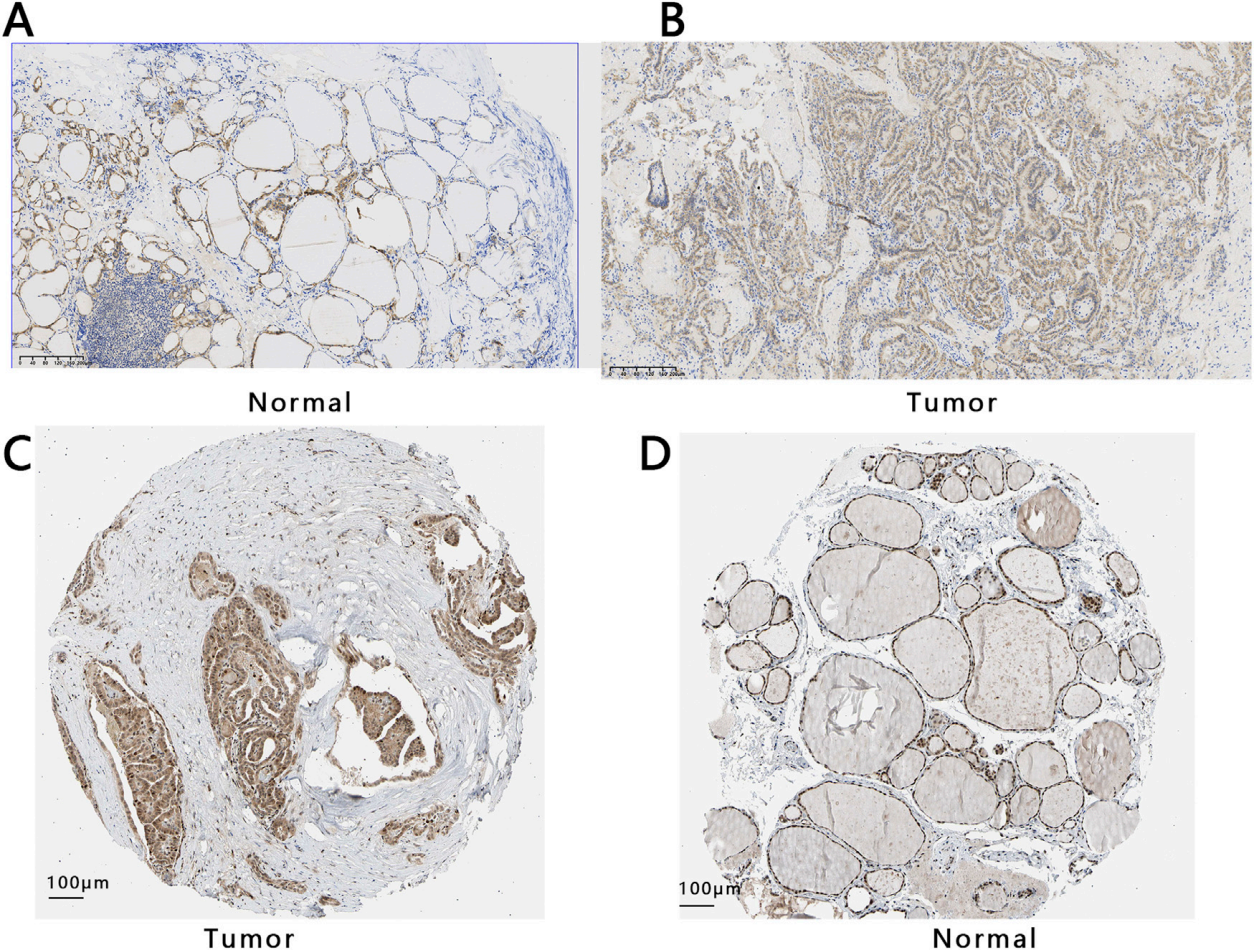
The protein expression of E2F1 in THCA and normal group. Expression analysis of E2F1 protein both in normal tissue **(A)** and PTC tissue **(B)** with a 200 μm ruler from collected tissues. Expression analysis of E2F1 protein in PTC tissue **(C)** and normal tissues **(D)** with a 100 μm ruler from the HPA database.

To study the expression of E2F1 in tumors, we performed three analyses in this work. The expression of E2F1 in the pan-cancer analysis indicated that it had a higher expression in tumors than in normal tissue (Figure 2A). The E2F1 expression was higher in PTC tumors than in normal (p < 0.05) based on the TCGA-PTC dataset (Figure 2B). Meanwhile, the paired t-test between the PTC tissue and its adjacent normal thyroid tissues also indicated the expression of E2F1 was higher in the tumor than in adjacent normal thyroid tissues (Figure 2D). We also verified the expression of this gene in the GEO database and obtained consistent results (Figures 2C,E). We further detected its protein expression in clinical PTC tissue and normal tissue, representative images as shown in Figures 3A,B, respectively. The results showed that the level of E2F1 protein was higher in PTC tissue than in normal thyroid tissue. In addition, we also verified the difference of protein E2F1 in both PTC tissue and normal tissue from the HPA database, and representative images were shown in Figures 3C,D, respectively.

### 3.4 Clinical value of E2F1 in patients with PTC

**FIGURE 4:**
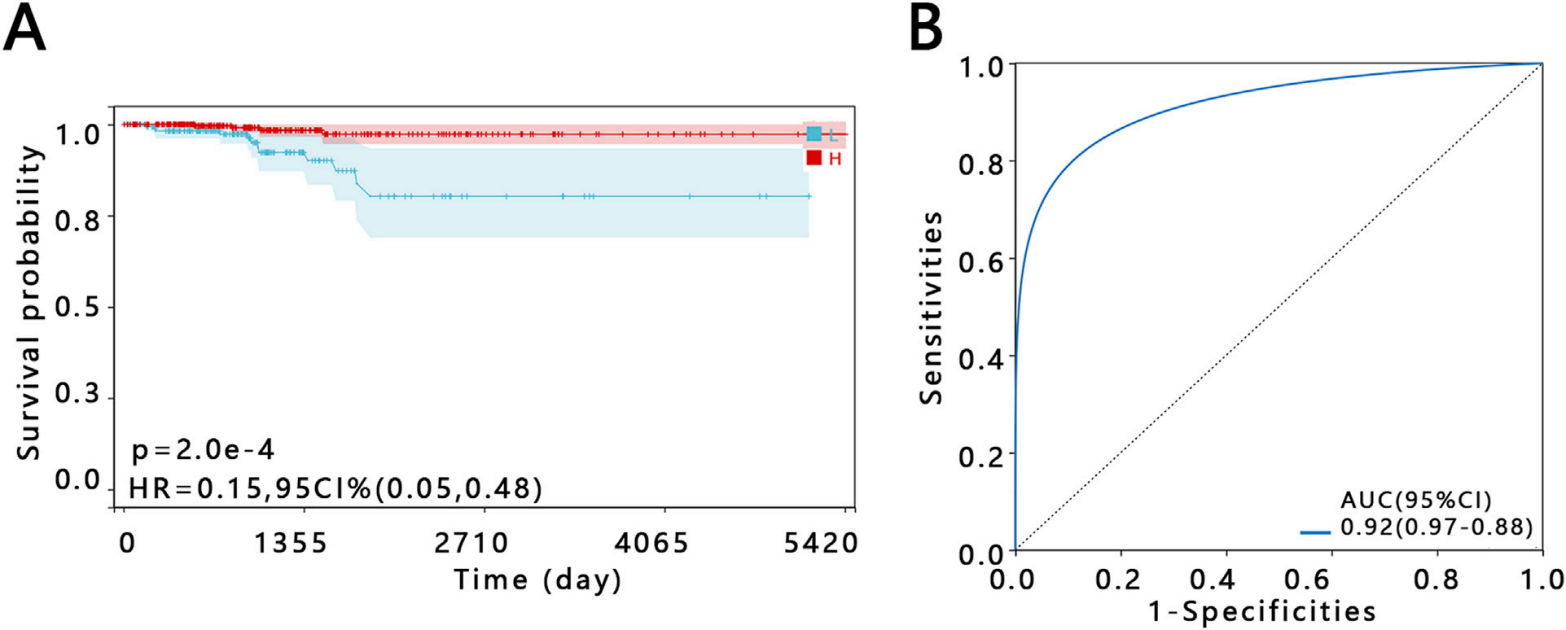
Prognostic value of E2F1 in PTC patient. Kaplan–Meier survival curve **(A)**. ROC curve **(B)**.

The relationship between the expression of E2F1 and the OS was analyzed based on TCGA database. The KM curve indicated that the expression of E2F1 impacted the survival of patients with PTC. The PTC patients with higher expression of the E2F1 had longer survival times (Figure 4A). Then, the ROC analysis was performed to distinguish the patients with PTC from individuals based on the expression of E2F1. The result of the area under the curve (AUC) for ROC was 0.92 and the 95% confidence interval (95% CI) was (0.97–0.88), which indicated that E2F1 had a good performance in the prediction of patient’s prognosis (Figure 4B).

### 3.5 The effect of E2F1 on PTC tumor cell senescence

**FIGURE 5:**
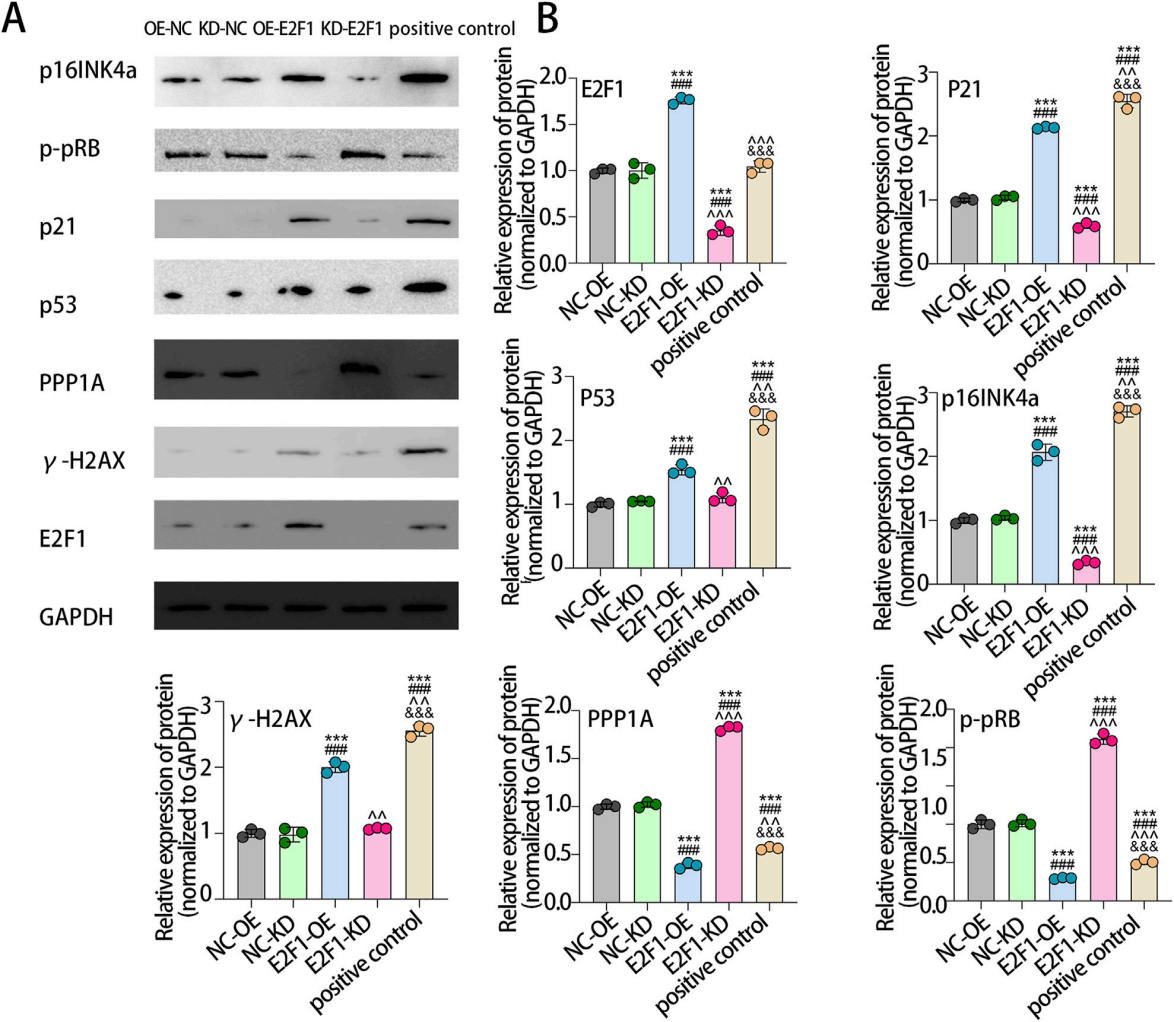
E2F1 affects tumor cell senescence and apoptosis. Western blot examination of cell senescence markers in each group **(A)**. Bar chart of the relative expression of protein. Note: The experiment was repeated three times **(B)**. * means that compared with NC-OE, there were significant statistical differences in other groups. # means that compared with NC-KD, there were significant statistical differences in other groups. ^ means that compared with E2F1-OE, there were significant statistical differences in other groups. & means that compared with E2F1-KD, there were significant statistical differences in other groups. Two symbols indicate p < 0.01. Three symbols indicate p < 0.001.

To elucidate the role of E2F1 in regulating cellular senescence in PTC, TPC-1 cells were subjected to different treatments. Senescence-associated markers were examined by Western blot analysis, while flow cytometry was employed to evaluate cell viability, thereby delineating the impact of E2F1 on tumor cell senescence. Five different treatment groups (knocked down E2F1 (E2F1-KD), upregulated E2F1 (E2F1-OE), NC-OE, NC-KD, positive control) were initially established, and Western blot analysis was performed to assess E2F1 protein expression across the groups. The results demonstrated that E2F1 protein levels were markedly elevated in the E2F1-OE group compared with the other groups. The E2F1 protein level in the E2F1-KD group was significantly lower than that in the other groups, indicating that the E2F1 overexpression or knockout treatment was successful. Western blot analysis further revealed that, compared with the NC-OE group, the E2F1-OE group exhibited significantly increased relative protein expression of p21, p53, γ-H2AX, and p16INK4a, while PPP1A and phosphorylated pRB (p-pRB) levels were markedly reduced. In contrast, compared with the NC-KD group, the knockdown of E2F1 resulted in significantly decreased expression of p21 and p16INK4a, accompanied by increased expression of PPP1A and p-pRB (Figures 5A,B). These results suggest that overexpression of the E2F1 gene promotes the senescence of tumor cells, and downregulation of the E2F1 gene inhibits the senescence of tumor cells.

**FIGURE 6:**
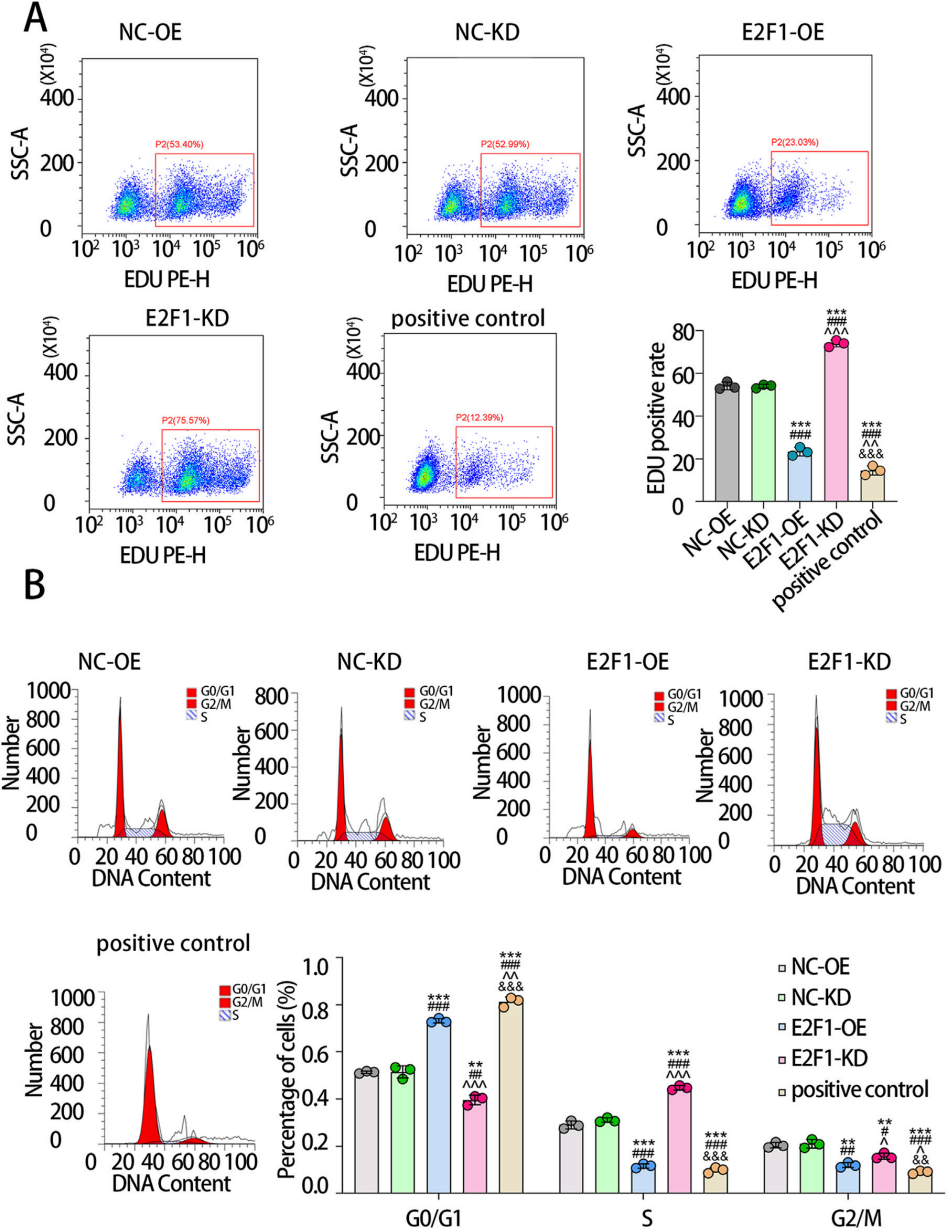
Flow cytometry analysis of senescent cells in different groups. The scatter plot displays the relative intensity of the EDU PH-E fluorescence signal on the ordinate against the side scatter (SSC) on the abscissa, with each point representing an individual cell. The percentage indicates the ratio of EDU-positive cells to the total population **(A)**. In the histogram, DNA content is plotted on the abscissa and cell count on the ordinate. The three peaks correspond sequentially to the G0/G1, S, and G2/M phases of the cell cycle **(B)**. Note: The experiment was repeated three times. P < 0.05 is considered statistically significant. * means that compared with NC-OE, there were significant statistical differences in other groups. # means that compared with NC-KD, there were significant statistical differences in other groups. ^ means that compared with E2F1-OE, there were significant statistical differences in other groups. & means that compared with E2F1-KD, there were significant statistical differences in other groups. A symbol indicates p < 0.05; Two symbols indicate p < 0.01. Three symbols indicate p < 0.001.

We further validated these findings using flow cytometry. The results showed that, compared with the NC-OE group, the proportion of EdU-positive cells was significantly reduced in the E2F1-OE group, whereas the E2F1-KD group exhibited a significantly higher proportion of EdU-positive cells relative to the NC-KD group (Figure 6A). Flow cytometric cell cycle analysis revealed that (Figure 6B), compared with the NC-OE group, the E2F1-OE group displayed a markedly increased proportion of cells in the G0/G1 phase, accompanied by a significant reduction in both S-phase and G2/M-phase cells. Notably, the majority of cells in the E2F1-OE group accumulated in the G0/G1 phase, indicating that E2F1 overexpression induced cell cycle arrest. In contrast, relative to the NC-KD group, the E2F1-KD group exhibited a significantly decreased proportion of G0/G1-phase cells, with a concomitant increase in S-phase cells and a reduction in G2/M-phase cells.

### 3.6 The effect of E2F1 on normal thyroid cells *in vitro*

**FIGURE 7:**
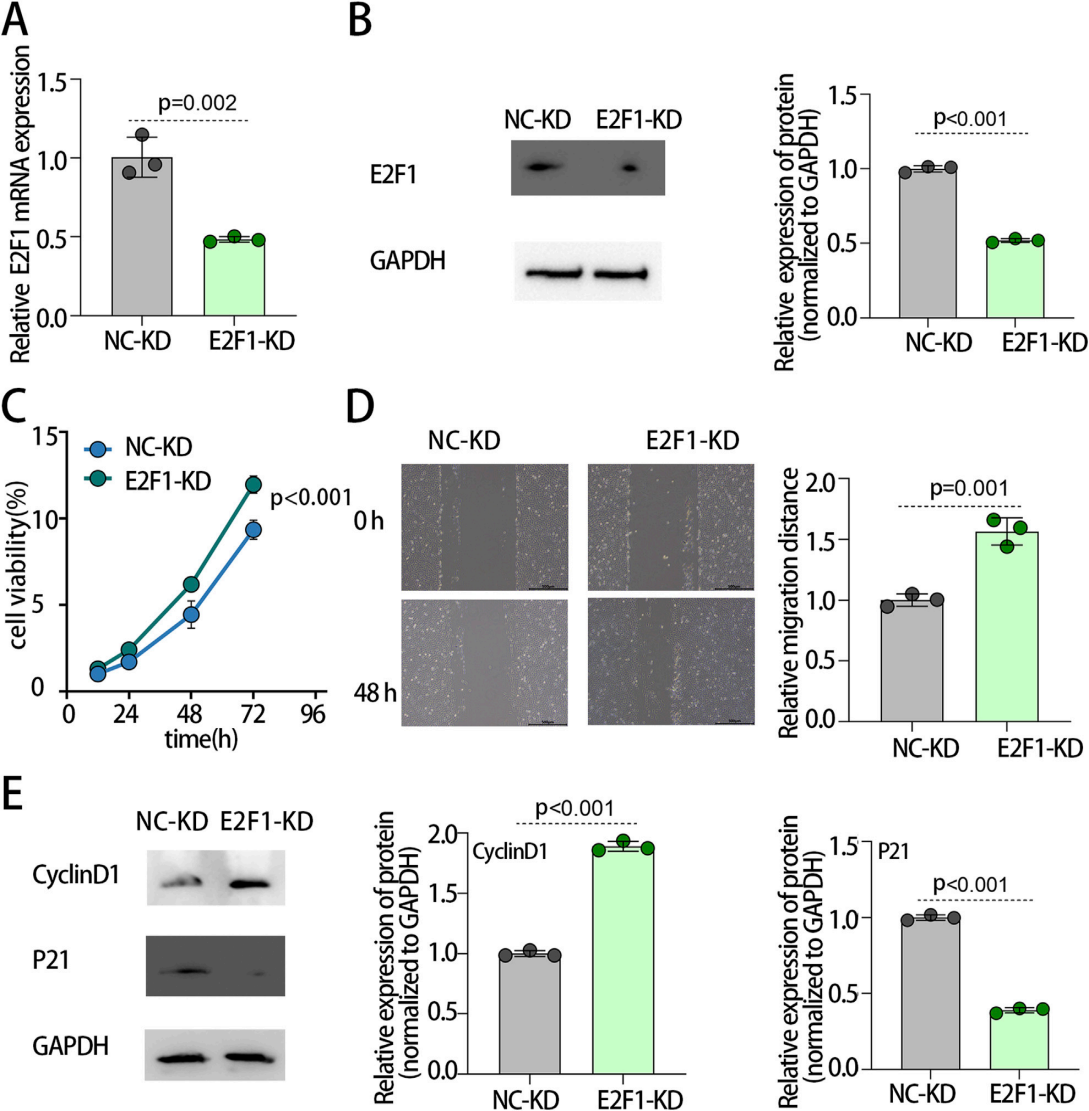
E2F1 knockdown promoted proliferation, invasion, and migration of normal thyroid cells *in vitro*. The mRNA expression level of E2F1 in normal thyroid cells by PCR **(A)**. The protein levels verification of E2F1 after downregulation treatment by Western blot **(B)**. The Cell viability detection by Cell Counting Kit-8 **(C)**. The detection of cell migration by the wound healing assay **(D)**. The protein levels of the cell cycle biomarker after downregulation treatment by Western blot **(E)**. Note: The experiment was repeated three times. p < 0.05 is considered statistically significant.

The above findings indicate that E2F1 suppresses tumor cell progression by promoting cellular senescence. Based on this, we hypothesized that reduced E2F1 expression in normal cells might facilitate their malignant transformation. To test this hypothesis, we downregulated E2F1 in normal human thyroid follicular epithelial cells (Nthy-ori 3-1) and assessed the resulting phenotypic changes. PCR analysis confirmed effective knockdown, showing a significant reduction in E2F1 mRNA levels in the E2F1-KD group compared with the NC-KD group (Figure 7A). Consistently, Western blot demonstrated a marked decrease in E2F1 protein expression in the E2F1-KD group relative to controls (Figure 7B). Functionally, CCK-8 assays revealed that E2F1 knockdown significantly enhanced cell viability (Figure 7C). Similarly, wound healing assays showed a pronounced increase in cell migratory distance following E2F1 downregulation (Figure 7D). Moreover, Western blot indicated that E2F1 knockdown elevated Cyclin D1 expression while reducing P21 levels (Figure 7E), supporting its role in promoting cell cycle progression and proliferation. Collectively, these results suggest that downregulated E2F1 in normal thyroid epithelial cells may drive malignant transformation by enhancing proliferative and migratory capacities.

### 3.7 The effect of E2F1 on PTC cells *in vitro*

**FIGURE 8:**
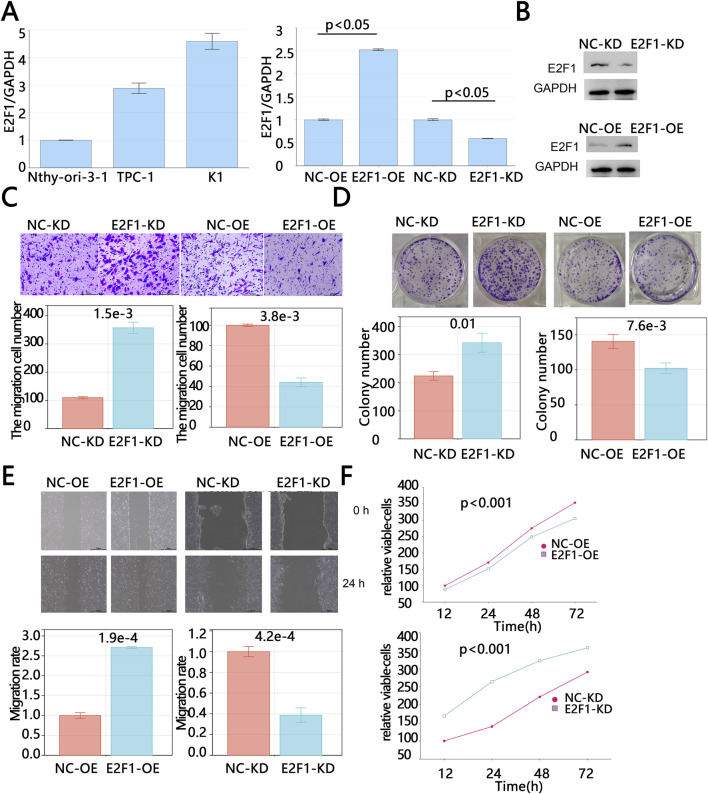
E2F1 knockdown promoted proliferation, invasion and migration of PTC *in vitro*. The baseline mRNA expression level of E2F1 in normal thyroid cells and cancer cells by PCR **(A)**. The mRNA and protein levels verification of E2F1 after upregulation and downregulation treatment **(B)**. The detection of cell invasion by Transwell assays **(C)**. The detection of cell colony formation by colony formation assays **(D)**. The detection of cell migration by wound healing assay **(E)**. The Cell viability detection by Cell Counting Kit-8 **(F)**. Note: The experiment was repeated three times. p < 0.05 is considered statistically significant.

The above results indicated that E2F1 was highly expressed in tumors, which was associated with improved prognosis for patients. To further investigate the impact of E2F1 on tumor progression, we conducted cell experiments. We compared the expression levels of E2F1 between normal thyroid cells (Nthy-ori-3-1) and thyroid cancer cells (TPC-1 and K1) via PCR. The PCR results revealed that E2F1 expression was higher in tumor cells compared to normal cells, with the expression level in K1 cells being higher than that in TPC-1 cells (Figure 8A). Therefore, the expression of E2F1 was knocked down in K1 cells and was upregulated in TPC-1 cells via lentivirus. RT-PCR and Western blot were used to evaluate the mRNA and protein levels of E2F1 expression after treatment, respectively. Figure 8B showed that the E2F1/GAPDH in the E2F1-OE group were higher than those in the vector group (p < 0.05); the E2F1/GAPDH in the E2F1-KD group were lower than those in the siRNA-NC group (p < 0.05), and Western blot has consistent results. Those results suggested that E2F1-KD cell models and E2F1-OE models were successfully constructed.

The results of the Transwell assay showed that the number of invasive cells in the E2F1-OE group was significantly lower than that in the vector-control group, while the number of invasive cells in the E2F1-KD group was significantly higher than that in the NC-KD group (Figure 8C). These findings suggest that the overexpression of E2F1 can inhibit tumor cell invasion. Consistent with this, scratch assay results demonstrated that E2F1 knockdown significantly increased the colony formation capacity of tumor cells (p < 0.05, Figure 8E). Colony formation assay results also showed that the number of colonies in the E2F1-OE group was significantly lower than that in the vector-control group, while the number of colonies in the E2F1-KD group was significantly higher than that in the NC-KD group (p < 0.05, Figure 8D), indicating that the upregulation of E2F1 can suppress tumor cell clonogenicity. Similarly, CCK-8 assay results demonstrated that tumor cell viability was significantly reduced following E2F1 overexpression, while E2F1 downregulation significantly increased tumor cell viability (p < 0.05, Figure 8F). In summary, the above results indicated that E2F1 upregulated dramatically inhibited the malignant proliferation, colony formation, and migration capacities of PTC cells *in vitro*.

### 3.8 GSEA

**FIGURE 9:**
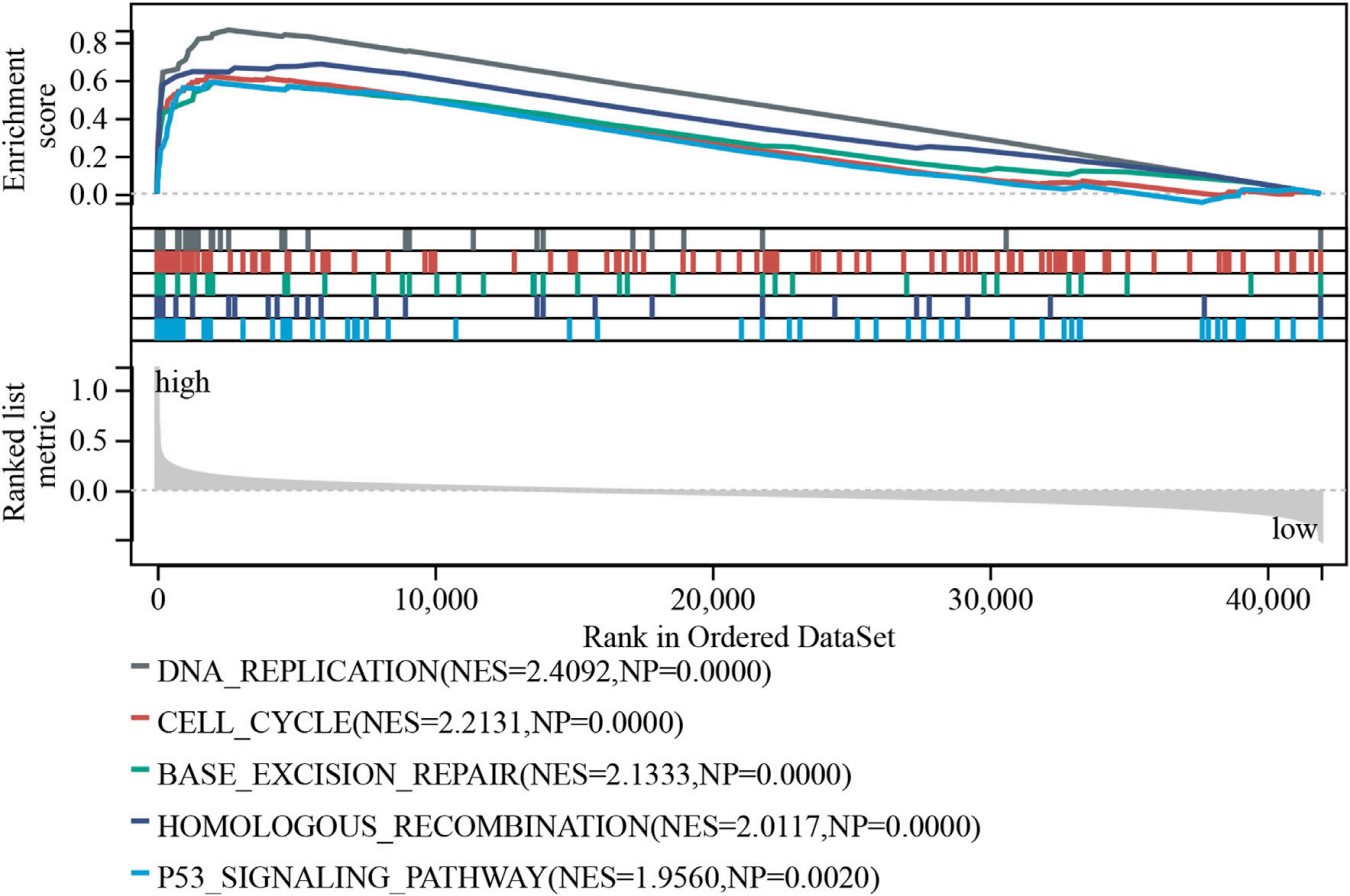
Exploration of E2F1 expression-related pathways. GSEA functional analysis in E2F1 low and high expression groups.

Patients with PTC were divided into two groups based on the cutoff values of the expression of E2F1. Then, the GSEA analysis was carried out to explore the related pathways associated with E2F1. The results showed that the high expression group of E2F1 was enriched in DNA replication, cell cycle, base excision repair, homologous recombination, and P53 signaling pathway (Figure 9).

### 3.9 Immune microenvironment and immune checkpoints correlation analysis

**FIGURE 10:**
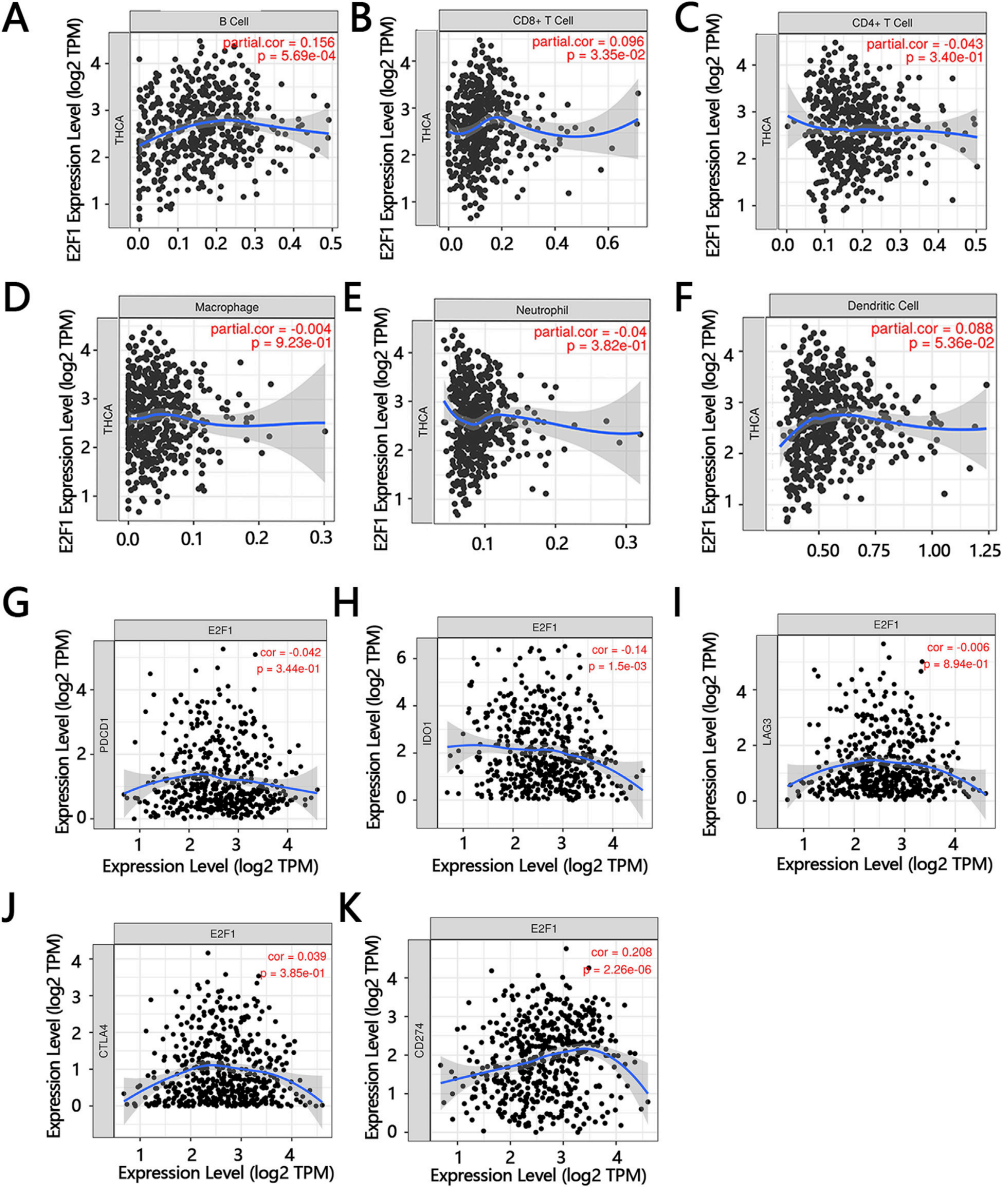
The relationship between E2F1 expression and immunity. The correlation between the expression of E2F1 and the infiltration level of B cell **(A)**, CD8^+^ T cell **(B)**, CD4^+^ T cell **(C)**, macrophage **(D)**, neutrophil **(E)**, dendritic cell **(F)**. The correlation of E2F1 with immune checkpoints including PDCD1 **(G)**, IDO1 **(H)**, LAG3 **(I)**, CTLA4 **(J)**, CD274 **(K)**.

The immune microenvironment plays an important role in the development of tumors. The results showed that there was a positive correlation between the expression of E2F1 and the infiltrating levels of three immune cells, including B cells, CD8+T cells, and dendritic cells (Figures 10A–F). Immune checkpoints are also related to the process of tumors by immune escape. The simple correlations between the E2F1 and main immune checkpoints were analyzed in this work. The results showed that E2F1 was negatively related to PDCD1, IDO1, and LAG3 (Figures 10G–I), while it was positively associated with CTLA4, and CD274 (Figures 10J,K).

### 3.10 Construction of nomogram

**FIGURE 11:**
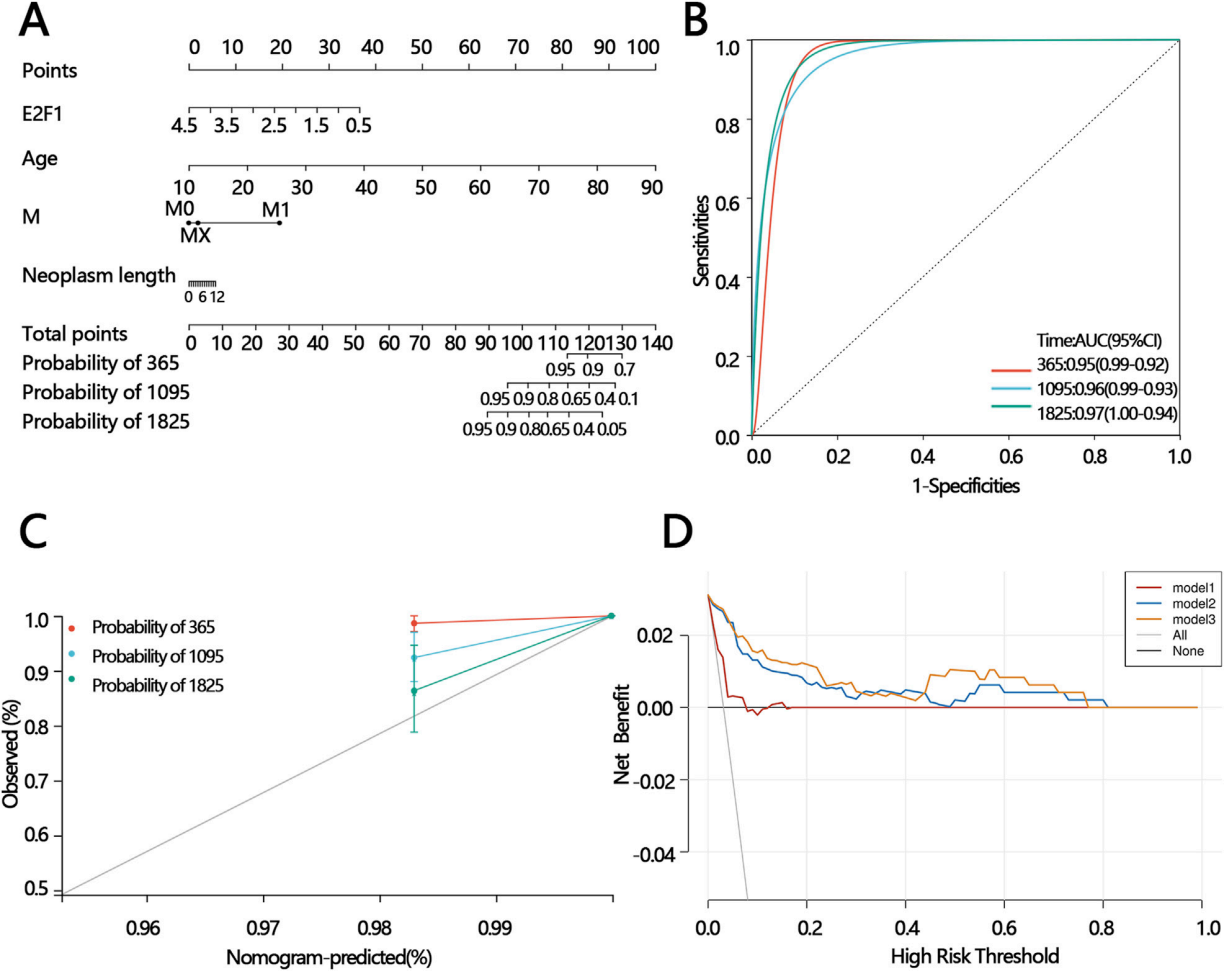
The construction of the prognostic model and prediction performance evaluation. The nomogram for predicting the proportion of patients with 365,1095,1825 days overall survival **(A)**. ROC analysis of the nomogram model **(B)**. Calibration curve of the nomogram model **(C)**. DCA of the nomogram models (mode 1: age; model 2: age and the expression of E2F1; model 3: M, the expression of E2F1, age, and neoplasm length) **(D)**.

To offer a precise model for predicting the personal prognosis of patients with PTC. The nomogram was constructed based on the results of univariable and multivariable Cox regression analysis. Finally, age, M, and neoplasm length were independent factors that affect survival (Table 2). Subsequently, those factors and E2F1 were united to construct the nomogram (Figure 11A). Next, three methods were used to assess the function of the nomogram. The AUC for the ROC curve was 0.95,0.96.0.97 at 365, 1,095, and 1825 days, respectively (Figure 11B). The calibration plots of the nomogram showed good agreement between the actual observations and the predicted ones (Figure 11C). The DCA curve showed greater net benefits across a range for the nomogram (Figure 11D).

**TABLE 2:** Cox regression analysis of E2F1 expression and clinical features in overall survival in patients.

Variables	Univariable		Multivariable	
	p	HR [95%CI]	p	HR [95%CI]
E2F1	0.007	0.346 [0.160,0.752]	0.880	0.117 [0.024,0.570]
Age	<0.001	1.161 [1.102,1.224]	<0.001	1.207 [1.112,1.310]
Sex group	0.207	1.942 [0.696,5.319]	0.862	0.873 [0.189,4.034]
T group	0.002	2.597 [1.405,4.797]	0.056	4.307 [0.966,19.198]
N group	0.241	1.533 [0.751,3.129]	0.340	0.469 [0.099,2.225]
M group	0.551	0.855 [0.511,1.431]	0.036	2.435 [1.060,5.594]
Stage	<0.001	2.409 [1.533,3.787]	0.751	1.284 [0.274,6.027]
Neoplasm depth	0.027	1.827 [1.027,3.114]	0.698	0.825 [0.312,2.180]
Neoplasm length	0.017	1.346 [1.055,1.718]	0.030	0.294 [0.097,0.889]
Neoplasm width	0.019	1.582 [1.077,2.323]	0.155	2.881 [0.670,12.377]

Abbreviation: CI: confidence interval; HR: hazard ratio.

## 4 Discussion

PTC with high occurrence seriously affects the health and life of people and increases the medical burden. Therefore, screening new prognosis biomarkers from the different research hotspots. Cell senescence was important for different tumor development. However, the relationship between the PTC prognosis and cell senescence was unclear. This paper screened the related prognosis cell senescence based on the different expression levels of genes between the normal and the patient with PTC. Then, we obtained that the cell senescence gene E2F1 was the key prognosis gene. However, the reported papers that related the correction between E2F1 and PTC were rare.

E2F1 is a main member of the E2F family. The E2F family plays an important role in cell cycle signal pathway and apoptosis (Buccitelli et al., 2017). In addition, it is also a target of the transforming proteins of small DNA tumor viruses (Martinez-Balbas et al., 2000). Its function is different in various tumors. The results of this work showed that the expression of E2F1 was higher in various tumors than in normal. Consistently, Ou et al. suggested the E2F1 expression was aberrantly upregulated in patients with acute myeloid leukemia, especially in those patients who had a mutation of FLT3-ITD (Ou et al., 2023).

E2F1 has important functions including DNA replication, checkpoint response and DNA repair in the cell cycle process in tumor cells, which has been widely reported in many studies (Manickavinayaham et al., 2020; Fouad et al., 2020). Moreover, the specific regulating process is different, and relates to different genes in various tumors. For instance, E2F1-mediated ectopic expression of PP1A promotes breast cancer progression via activation of YAP1 (Deng et al., 2023). Wang et al. found that the Rb-E2F1 axis which was a crucial compound in the cell cycle was regulated by CENPF to improve the drug sensitivity in chemotherapy of triple-negative breast cancer (Wang et al., 2023). Collectively, these studies reported that E2F1 was an oncogene. However, the survival analysis results indicated that E2F1 high expression was beneficial to the survival of patients with PTC in this study. In our cellular experiments, we found that the overexpression of E2F1 inhibited tumor cell invasion, migration, activity, and colony formation. We speculate that elevated E2F1 increases replication throughput, provoking replication stress and a DNA damage response (DDR) in which E2F1 is stabilized via the ATM/ATR–CHK2 pathway and undergoes a functional switch from pro-proliferative driver to damage sensor/effector (Lin et al., 2001; Zannini et al., 2014). In this context, E2F1 stabilizes p53 through the p14ARF–MDM2 axis and upregulates p21, or engages the p16–RB pathway, thereby triggering irreversible cell-cycle arrest and senescence that directly restrains tumor-cell proliferation (Dimri et al., 2000). This model is concordant with our GSEA data, E2F1 not only transcriptionally upregulates homologous recombination (HR) genes such as BRCA1 and RAD51, enhancing repair/checkpoint capacity, but also recruits repair complexes to sites of damage in a non-transcriptional manner, functionally coupling high replication flux to high-fidelity repair (Wang et al., 2000; Chen et al., 2011; Orhan et al., 2021). When replication stress persists beyond the repair threshold, sustained activation of the p53-p21 and p16-RB axes drives a stable senescent state—consistent with our observations that E2F1 overexpression induces senescence in TPC-1 cells, whereas E2F1 knockdown in normal thyroid cells facilitates neoplastic transformation. Importantly, the overall low TP53 mutation burden and relative preservation of p53 function in PTC bias E2F1 toward the “DDR-senescence” trajectory rather than a pro-proliferative program, providing a mechanistic rationale for its association with favorable prognosis (McFadden et al., 2014).

E2F1-driven senescence also reshapes the tumor microenvironment via the SASP to limit metastatic potential. On the one hand, senescence rewires cytoskeletal, adhesion, and motility programs, intrinsically reducing migratory and invasive capacities; on the other, SASP factors (e.g., IL-6/IL-8 and chemokines) enhance immune surveillance and phagocytic clearance, creating—when appropriately timed and sufficiently deep—a tumor-suppressive ecosystem that scales single-cell arrest into population-level constraints on dissemination (Chibaya et al., 2022). In line with this, we found that high E2F1 expression correlates positively with B cells, CD8^+^ T cells, and dendritic cells, suggesting that SASP-linked chemokines (such as CCL5 and CXCL9/10) and interferon signaling promote antigen presentation and effector T-cell recruitment, thereby amplifying “senescence surveillance” and restraining tumor growth and spread—findings that dovetail with the observed suppression of malignant phenotypes and improved prognosis in the E2F1-high setting (Chibaya et al., 2022; Chen et al., 2023).

## 5 Strengths and limitations

This study has several strengths: (1) It is the first to identify the tumor-suppressive role of the senescence-associated E2F1 gene in PTC. This finding contradicts the majority of previously reported studies (Rocca et al., 2019; Gonzalez-Romero et al., 2021; Li et al., 2023) that conclude E2F1 as an oncogene, providing a reference for further investigation into the varying roles of E2F1 in different cancers. (2) This study combines bioinformatics analysis and experimental verification to preliminarily explore and explain the potential mechanisms underlying the inconsistency with previously reported research findings. However, the study also has some limitations as follows. (1) We only verified the relationship between E2F1 and PTC cell senescence, and E2F1 is related to the malignant phenotype of PTC. Although we speculated on the potential mechanism among E2F1, cell senescence, and malignant phenotype of PTC based on reported studies, we did not conduct *in vitro* and *in vivo* experiments to verify a specific mechanism (such as the role of immune cells in promoting tumor cell metastasis through low expression of E2F1) due to limited conditions. (2) Cellular experiments only used two PTC cell lines, which may lead to result bias. (3) Immune cell infiltration was assessed exclusively through the TIMER database, without validation via flow cytometry or immunofluorescence assays. Although this study has its limitations, it provides a reference for subsequent research on the role of the cellular senescence gene in PTC and also provides potential target genes for the precise treatment of PTC.

## 6 Conclusion

In the present work, we found that the cell senescence-related gene E2F1 was upregulated in PTC and its high expression was significantly associated with the favorable prognosis of patients. E2F1 can inhibit the development of malignant tumor phenotypes by regulating cellular senescence, thereby reducing the risk of poor prognosis. The model based on E2F1 and clinical features can accurately predict poor prognosis in patients, and E2F1 may serve as a potential therapeutic target for PTC.

## Data Availability

The datasets used and/or analyzed during the current study available from the corresponding author on reasonable request.
