# Supplementary material for: Identification of prognostic and cellular senescence gene E2F1 of papillary thyroid carcinoma through bioinformatics analyses and experimental verification
Source: Front Genet. 2025 Sep 18;16:1605385. doi: 10.3389/fgene.2025.1605385 (PMC12488402; doi:10.3389/fgene.2025.1605385)
Supplement: Supplementary file 2 [file DataSheet1.docx]

| **Supplementary Table S1** cellular senescence gene set obtained from the website of CellAge | | | |
| --- | --- | --- | --- |
| Gene | Gene | Gene | Gene |
| AAK1 | ID1 | PPM1B | TLR3 |
| ABI3 | ID4 | PPM1D | TMSB4X |
| ACLY | IFNG | PRKCD | TNFSF13 |
| ADCK5 | IGFBP1 | PRKCH | TNFSF15 |
| AGT | IGFBP3 | PRMT6 | TOP1 |
| AKR1B1 | IGFBP5 | PROX1 | TP53 |
| AKT1 | IGFBP6 | PRPF19 | TP63 |
| ALOX15B | IL1A | PSMB5 | TPR |
| AR | ING1 | PSMD14 | TRIM28 |
| ARPC1B | ING2 | PTTG1 | TRPM8 |
| ASF1A | IRF3 | RAD21 | TXN |
| ASPH | IRF5 | RAF1 | TXNIP |
| ATF7IP | IRF7 | RB1 | TYK2 |
| ATM | ITGB4 | RBP2 | UBTD1 |
| AURKA | ITPK1 | RBX1 | USP1 |
| AXL | ITPKB | RNASEL | VEGFA |
| BAG3 | ITSN2 | RPS6KA6 | VENTX |
| BCL6 | KCNJ12 | RSL1D1 | WNT16 |
| BHLHE40 | KDM4A | RUNX1 | WNT2 |
| BLK | KDM5B | RUVBL2 | WRN |
| BLVRA | KIAA1524 | SELENOH | WT1 |
| BMI1 | KL | SENP1 | WWP1 |
| BRAF | KSR2 | SENP2 | XAF1 |
| BRCA1 | LATS1 | SENP7 | YAP1 |
| BRD7 | LEO1 | SERPINE1 | YPEL3 |
| BTG3 | LGALS3 | SFN | ZFP36 |
| CAV1 | LIMA1 | SGK1 | ZMAT3 |
| CAVIN1 | LIMK1 | SIK1 | ZNF148 |
| CBX7 | MAD2L1 | SIN3B | FASTK |
| CBX8 | MAGEA2 | SIRT1 | FBXO31 |
| CCND1 | MAGOH | SIRT6 | FOS |
| CDK1 | MAGOHB | SIX1 | FOXM1 |
| CDK18 | MAP2K1 | SLC13A3 | FOXO3 |
| CDK2AP1 | MAP2K2 | SLC16A7 | FXR1 |
| CDK4 | MAP2K3 | SMARCA4 | G6PD |
| CDK6 | MAP2K6 | SMARCB1 | GAPDH |
| CDKN1A | MAP2K7 | SMG1 | GATA4 |
| CDKN1B | MAP3K6 | SMURF2 | GKN1 |
| CDKN1C | MAP3K7 | SNAI1 | GLB1 |
| CDKN2A | MAP4K1 | SOCS1 | GNG11 |
| CDKN2AIP | MAPK12 | SOD1 | GRK6 |
| CDKN2B | MAPK14 | SORBS2 | OTX2 |
| CEBPB | MAPKAPK5 | SOX2 | P3H1 |
| CENPA | 44990 | SOX5 | PAK4 |
| CHEK1 | MAST1 | SP1 | PATZ1 |
| CKB | MATK | SPIN1 | PBRM1 |
| CPEB1 | MCL1 | SPOP | PCGF2 |
| CSNK1A1 | MCRS1 | SRC | PDCD10 |
| CSNK2A1 | MDH1 | SREBF1 | PDIK1L |
| CTNNAL1 | MECP2 | SRSF1 | PDPK1 |
| CXCL1 | MMP9 | STAT5B | PDZD2 |
| CXCL8 | MOB3A | STK32C | PEBP1 |
| CYR61 | MORC3 | STK40 | PEX19 |
| DDB2 | MORF4 | SUPT5H | PIAS4 |
| DEK | MVK | SYK | POT1 |
| DGCR8 | MXD4 | TACC3 | POU5F1 |
| DHCR24 | MYC | TBX2 | HDAC1 |
| DHX9 | MYLK | TERC | HDAC4 |
| DLX2 | NADK | TERF2 | HEPACAM |
| DPY30 | NANOG | TERT | HIVEP1 |
| DUSP16 | NDRG1 | TFAP4 | HJURP |
| DUSP3 | NEK1 | TFDP1 | HK3 |
| E2F1 | NEK4 | TGFB1I1 | HMGB1 |
| EHF | NEK6 | PIK3C2A | HRAS |
| ENDOG | NFE2L2 | PIK3R5 | HSPA5 |
| EPHA3 | NINJ1 | PIM1 | HSPB2 |
| ERRFI1 | NOTCH3 | PKM | PNPT1 |
| ETS1 | NOX4 | PLA2R1 | NUAK1 |
| ETS2 | NR2E1 | PML | EZH2 |
| EWSR1 | NTN4 | PMVK |  |

| **Supplementary Table S2** DECSG set obtained by “limma” R package | | | | | |
| --- | --- | --- | --- | --- | --- |
| Upregulated gene | logFC | adj p | Downregulated gene | logFC | adj p |
| SREBF1 | 1.074 | <0.001 | PLA2R1 | -2.761 | <0.001 |
| TMSB4X | 1.110 | <0.001 | SORBS2 | -2.048 | <0.001 |
| MAP3K6 | 1.112 | <0.001 | FOS | -1.882 | <0.001 |
| CDKN1A | 1.236 | <0.001 | EPHA3 | -1.640 | <0.001 |
| CDKN2B | 1.241 | <0.001 | ID4 | -1.543 | <0.001 |
| E2F1 | 1.248 | <0.001 | PROX1 | -1.437 | <0.001 |
| RUNX1 | 1.297 | <0.001 | SNAI1 | -1.289 | <0.001 |
| ITGB4 | 1.311 | <0.001 | RPS6KA6 | -1.171 | <0.001 |
| ZMAT3 | 1.378 | <0.001 |  |  |  |
| BHLHE40 | 1.414 | <0.001 |  |  |  |
| CCND1 | 1.623 | <0.001 |  |  |  |
| IGFBP6 | 1.653 | <0.001 |  |  |  |
| ALOX15B | 2.013 | <0.001 |  |  |  |
| LGALS3 | 2.119 | <0.001 |  |  |  |
| SFN | 2.338 | <0.001 |  |  |  |

| **Supplementary Table S3** PCSG set obtained by univariate COX regression | | | | |
| --- | --- | --- | --- | --- |
| gene | Hazard Ratio | Lower | Upper | p |
| SNAI1 | 2.907 | 1.871 | 4.516 | 0.000 |
| TERT | 6.741 | 1.775 | 25.595 | 0.005 |
| WT1 | 9.245 | 2.228 | 38.358 | 0.002 |
| NDRG1 | 8.066 | 2.644 | 24.606 | 0.000 |
| MAST1 | 5.340 | 1.676 | 17.016 | 0.005 |
| PIM1 | 2.559 | 1.441 | 4.546 | 0.001 |
| NINJ1 | 0.194 | 0.067 | 0.568 | 0.003 |
| SOX2 | 847.246 | 6.692 | 107267.278 | 0.006 |
| HDAC4 | 7.440 | 1.894 | 29.217 | 0.004 |
| CDKN2A | 1.869 | 1.183 | 2.952 | 0.007 |
| E2F1 | 0.344 | 0.159 | 0.746 | 0.007 |
| IL1A | 2.635 | 1.186 | 5.853 | 0.017 |
| NEK1 | 7.431 | 1.458 | 37.875 | 0.016 |
| CXCL1 | 1.706 | 1.075 | 2.705 | 0.023 |
| MAP3K6 | 0.404 | 0.183 | 0.893 | 0.025 |
| PLA2R1 | 1.649 | 1.052 | 2.584 | 0.029 |
| ASPH | 2.485 | 1.159 | 5.329 | 0.019 |
| PRKCD | 0.207 | 0.056 | 0.762 | 0.018 |
| SERPINE1 | 1.443 | 1.027 | 2.026 | 0.034 |
| HMGB1 | 0.243 | 0.068 | 0.868 | 0.029 |
| LGALS3 | 0.759 | 0.580 | 0.993 | 0.044 |
| UBTD1 | 0.315 | 0.106 | 0.938 | 0.038 |
| P3H1 | 3.030 | 1.065 | 8.619 | 0.038 |
| WWP1 | 4.746 | 1.076 | 20.927 | 0.040 |
| DDB2 | 0.405 | 0.166 | 0.989 | 0.047 |
